# Multicellular Bioprinting of Biomimetic Inks for Tendon‐to‐Bone Regeneration

**DOI:** 10.1002/advs.202301309

**Published:** 2023-04-29

**Authors:** Lin Du, Chen Qin, Hongjian Zhang, Fei Han, Jianmin Xue, Yufeng Wang, Jinfu Wu, Yin Xiao, Zhiguang Huan, Chengtie Wu

**Affiliations:** ^1^ State Key Laboratory of High Performance Ceramics and Superfine Microstructure Shanghai Institute of Ceramics Chinese Academy of Sciences 1295 Dingxi Road Shanghai 200050 P. R. China; ^2^ Center of Materials Science and Optoelectronics Engineering University of Chinese Academy of Sciences 19A Yuquan Road Beijing 100049 P. R. China; ^3^ Nanjing First Hospital Nanjing Medical University 68th Changle Road Nanjing Jiangsu 210006 P. R. China; ^4^ School of Medicine and Dentistry Menzies Health Institute Queensland Griffith University Queensland 4222 Australia

**Keywords:** 3D bioprinting, biomaterials, biomimetic, tendon‐to‐bone interface regeneration

## Abstract

Tendon‐to‐bone interface has a hierarchical structure and gradient component that are conducive to distributing the stresses to achieve movement. Conventional biomaterials lack the capacity to induce synchronous repair of multiple tissues, resulting in the failure of the interface repair. Biomimetic strategies have attracted enormous attention in the field of complex structure regeneration because they can meet the different physiological requirements of multiple tissues. Herein, a biomimetic ink mimicking tendon/bone tissues is developed by combining tendon/bone‐related cells and Mo‐containing silicate (MS) bioceramics. Subsequently, biomimetic multicellular scaffolds are fabricated to achieve the simulation of the hierarchical structure and cellular composition of tendon‐to‐bone interfaces by the spatial distribution of the biomimetic inks via 3D bioprinting, which is of great significance for inducing the regeneration of complex structures in the interface region. In addition, attributed to the desirable ionic microenvironment created by MS bioceramics, the biomimetic scaffolds possess the dual function of inducing tendon/bone‐related cells tenogenic and osteogenic differentiation in vitro, and promote the integrated regeneration of tendon‐to‐bone interfaces in vivo. The study offers a feasible strategy to construct biomimetic multicellular scaffolds with bifunction for inducing multi‐lineage tissue regeneration, especially for regenerating soft‐to‐hard tissue interfaces.

## Graphical abstract

A biomimetic multicellular scaffold for the functional regeneration of tendon‐to‐bone interfaces is developed by combining multiple cells and Mo‐containing silicate bioceramics via 3D bioprinting technology. The biomimetic scaffolds simulate the hierarchical structure and cellular composition of tendon‐to‐bone interfaces and can promote the integrated regeneration of tendon‐to‐bone interface.

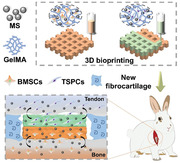

## 1 Introduction

Soft and hard tissue damage usually occurs simultaneously in severe bone defects, cartilage damage, teeth missing, or tendon injury.^[^ ^1^ ^]^ At present, integrated regeneration of soft/hard composite tissues remains an intractable problem in clinics, since the frequent failure of reconstructing complex soft to hard tissue interfaces.^[^ ^2^ ^]^ For example, half of the patients who suffer tendon injury are faced with recidivation after surgery due to the poor healing rate of the tendon‐to‐bone interface.^[^ ^3^ ^]^ Tendon‐to‐bone interface, composed of the tendon, bone, and transitional fibrocartilage, has a complex hierarchical structure including gradient mineral ingredient, extracellular matrix, and cell phenotype.^[^ ^4^ ^]^ Traditional surgery tends to form disorganized fibrovascular scar tissue, resulting in poor interfacial structures and weakened mechanical strength.^[^ ^5^ ^]^ Although various biomaterials, such as polyethylene terephthalate, polyglycolic acid, and hydroxyapatite, have been applied to treat tendon‐to‐bone injury, the lack of bioactivity still impedes them from achieving satisfactory therapeutic outcomes.^[^ ^6^ ^]^ Researchers have employed growth factors to impart bioactivity to biomaterials, but such an approach can only promote partial tissue regeneration, namely either tendon or bone of the integration region.^[^ ^7^ ^]^ Recently, multicellular tissue engineering scaffolds based on biomimetic strategy have attracted much attention in tissue regeneration.^[^ ^8^ ^]^ These multicellular scaffolds could accurately recapitulate the multiple cellular distributions, hierarchical structure, and composition of targeted tissues through 3D bioprinting technology.^[^ ^9^ ^]^ Compare to the hydrogel scaffolds that only contain cells of soft/hard tissues,^[^ ^10^ ^]^ these multicellular scaffolds with multi‐level biomimetic features exhibit greater potential for the regeneration of complex tissues. Therefore, such a biomimetic multicellular strategy inspires the regeneration of the tendon‐to‐bone interface.

The design of bioinks is essential for biomimetic regeneration of tendon‐to‐bone interfaces. An ideal biomimetic ink can not only simulate the tissue microenvironment, but also modulate the activities of multiple cells to achieve desirable biological functions. Our previous studies have found that the specific ionic microenvironment built by silicate bioceramics could regulate the proliferation, adhesion, and specific differentiation of multiple cells in a multicellular system and promote functional tissue regeneration.^[^ ^11^ ^]^ In addition, molybdenum (Mo), an essential trace element of human body, is an important composition of various enzymes required for organismal metabolism and homeostasis.^[^ ^12^ ^]^ Some studies have found that Mo ions can stimulate osteogenic differentiation of BMSCs by enhancing alkaline phosphatase (ALP) expression and calcium nodules.^[^ ^13^ ^]^ Besides, it has been proved that Mo ions can induce the secretion of collagen fibers and enhance mitochondrial function.^[^ ^13^, ^14^ ^]^ Although, to our knowledge, the effect of Mo ions on tendon regeneration has not been investigated, it has been reported that promoting the formation of collagen fibers and strengthening of mitochondrial function is beneficial to tendon repair.^[^ ^15^ ^]^ Thus, Mo ions which can regulate mitochondrial function and secrete collagen may have some positive effects on tendon regeneration. Hence, based on this dual induction function of Mo ions, combined with the unique bioactivity of silicate bioceramics, we speculate that Mo‐containing silicate bioceramics can act as stable biological factors to simultaneously regulate the processes of osteogenesis and tendon formation, thereby promoting integrated regeneration of the tendon‐to‐bone interface.

Herein, we successfully developed a biomimetic ink composed of tendon stem/progenitor cells (TSPCs)/bone marrow mesenchymal stem cells (BMSCs) and Mo‐containing silicate (MS) bioceramics. These biomimetic inks could support cell survival and stimulate multiple cellular specifical differentiation. Then, a biomimetic multicellular scaffold mimicked the hierarchical structure and cellular composition of tendon‐to‐bone interfaces was fabricated by organizing tendon/bone‐related biomimetic inks in an orderly way using 3D bioprinting. Furthermore, MS bioceramics were added into bioinks as bio‐factors to achieve diverse bioactivities in inducing the migration and specific differentiation of multiple cells within the scaffolds. The positive impact of biomimetic multicellular scaffolds on osteogenic and tenogenic differentiation in vitro was demonstrated. Moreover, the function of Mo ions in promoting the bi‐directional activities of the multicellular scaffolds was thoroughly investigated. More importantly, the multicellular scaffolds successfully facilitated the integrated regeneration of tendon‐to‐bone interfaces after implanting into rabbit rotator cuff tear (RCT). Such biomimetic multicellular scaffolds offered an effective approach for the regeneration of soft‐to‐hard tissue interfaces.

## 2 Results and Discussion

### 2.1 Preparation and Characterization of Mo‐Containing Silicate (MS) Bioceramics and MS‐Containing Bioinks

**Figure 1:**
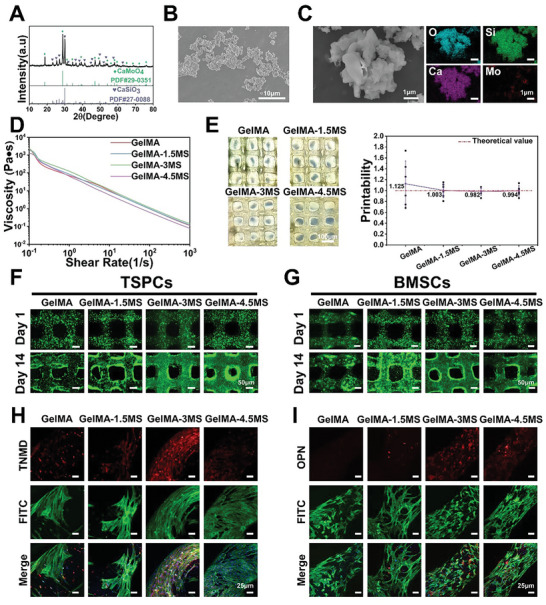
Characterization of Mo‐containing silicate (MS) bioceramics, MS‐containing bioinks, and the printed scaffolds with tissue cells. A) XRD pattern of MS particles. B) SEM image of MS particles. C) EDS elemental mapping of MS particles. D) The shear‐thinning properties of bioinks with different contents of MS particles. E) Optical photographs of 3D printed hydrogel scaffolds with different contents of MS particles, and corresponding printability values (*n* = 6). Live/dead assay of 3D bioprinted. F) TSPCs‐laden scaffolds and G) BMSCs‐laden scaffolds after cultured for 1 and 14 days. H) Immunofluorescence staining images of TNMD protein expression of TSPCs in 3D bioprinted TSPCs‐laden scaffolds after 14 days of culture. I) Immunofluorescence staining images of OPN protein expression of BMSCs in 3D bioprinted BMSCs‐laden scaffolds after 14 days of culture. MS‐containing bioinks could not only support the long‐term survival of both TSPCs and BMSCs, but also up‐regulate their expression level of specific differentiation markers.

Mo‐containing silicate bioceramics were prepared by a typical chemical coprecipitation method. Mo‐containing silicate bioceramics with various Mo contents (relative to the content of Ca) were synthesized by changing the molar percentage of H_24_Mo_7_N_6_O_24_·4H_2_O. They were denoted as CS (pure silicate), 2Mo‐CS (silicate with 2% Mo), 5Mo‐CS (silicate with 5% Mo), and 10Mo‐CS (silicate with 10% Mo). First, X‐ray diffraction (XRD) pattern showed that CS bioceramics were indexed into CaSiO_3_ phase with high crystallinity and purities (PDF No. 27‐0088). Meanwhile, the diffraction peaks of Mo‐containing silicate bioceramics could be indexed into CaSiO_3_ phase and CaMoO_4_ phase, indicating that the Mo element mainly existed as the form of CaMoO_4_ (PDF No. 29‐0351) (Figure S1, Supporting Information). Moreover, X‐ray fluorescence spectrometry (XRF) was used to measure the molar ratio of Mo/Ca in Mo‐containing silicate bioceramics. As shown in Table S1, Supporting Information, the molar ratio of Mo/Ca was 1.71%, 4.98%, and 7.55% in 2Mo‐CS, 5Mo‐CS, and 10Mo‐CS, respectively. As a result, Mo‐containing silicate bioceramics were successfully synthesized and 10Mo‐CS were selected for further experiments, denoted as MS (**Figure** **1**A). Observed by scanning electron microscope (SEM), MS particles were below 10 µm in diameter with irregular morphology (Figure 1B). In addition, energy dispersive spectroscopy (EDS) mapping demonstrated that Ca, O, Si, and Mo were uniformly distributed in the particle (Figure 1C).

To serve as the bioink matrix, gelatin methacrylate (GelMA) was synthesized according to previous studies.^[^ ^16^ ^]^ SEM found its interconnected macroporous structure, which was beneficial to nutrient delivery (Figure S2A, Supporting Information). Then, according to the mass ratio of MS bioceramics to freeze‐dried GelMA foam, MS bioceramics were added to GelMA matrix to form GelMA‐nMS (*n* = 0, 1.5, 3, 4.5) composite inks. The SEM and EDS mapping images of their cross‐section indicated that MS particles were evenly distributed inside the hydrogels and had no apparent effects on their macroporous structure (Figure S2B,C, Supporting Information). Besides, rheological analysis confirmed that the addition of MS did not affect the shear‐thinning performance of GelMA hydrogels (Figure 1D). Subsequently, the printability of these inks was evaluated by preparing a grid‐like structure. According to the optical images, it was found that the grid‐like structure of GelMA containing MS particles was squarer than pure GelMA. Meanwhile, the printability value of these inks was calculated to confirm that MS particle incorporation could increase the printability of GelMA inks (Figure 1E).

TSPCs are derived from tendon tissue and exhibit good proliferation and differentiation potential compared to other tenocytes.^[^ ^17^ ^]^ Due to these advantages, TSPCs have been used to construct bioprinting scaffolds.^[^ ^18^ ^]^ BMSCs, ideal candidates for bone tissue engineering, have been widely applied to construct biomimetic bone tissue scaffolds.^[^ ^19^ ^]^ Therefore, TSPCs and BMSCs were chosen as representative cells for tendon and bone in this work. First, TSPCs and BMSCs were incorporated into the composite inks to fabricate 3D bioprinted cell‐laden scaffolds. After that, the live/dead assay was conducted to evaluate the biocompatibility of GelMA‐MS bioinks. Both of TSPCs and BMSCs were uniformly distributed within the scaffolds and maintained a high survival rate during the culture periods (Figure 1F,G and Figure S3A,B, Supporting Information). In addition, Mo and Si ions were continuously released from 3D bioprinted scaffolds containing MS during the culture (Table S2, Supporting Information). Moreover, an immunofluorescent protein staining assay was performed to evaluate the effect of MS on regulating cell differentiation. The expression of TNMD protein, a transmembrane glycoprotein marker of tenocyte maturation, was detected in TSPCs.^[^ ^20^ ^]^ As shown in Figure 1H and Figure S3D, Supporting Information, the TNMD expression level of GelMA‐MS groups was significantly higher than GelMA groups, among which GelMA‐3MS groups had the highest expression level. Besides, the expression of OPN, one of the osteogenesis‐related proteins that play an important role in bone formation and remodeling,^[^ ^21^ ^]^ was tested in BMSCs. Figure 1I showed that much more OPN protein of BMSCs was expressed in GelMA‐MS groups as compared with GelMA groups, indicating that MS bioceramics had a promotion effect on the osteogenic differentiation of BMSCs. The semi‐quantitative analysis further demonstrated that the expression level of OPN in GelMA‐3MS and GelMA‐4.5MS groups was significantly higher than that in GelMA and GelMA‐1.5MS groups (Figure S3C, Supporting Information). Taken together, the above results indicated that MS‐containing bioinks had satisfactory bioactivities to support the survival and specific differentiation of encapsulated cells. Notably, the GelMA‐3MS group had the best capacity to promote the osteogenic differentiation of BMSCs and the tenogenic differentiation of TSPCs. Hence, GelMA‐3MS bioinks were chosen for further studies.

### 2.2 Preparation and Characterization of Biomimetic Multicellular Scaffolds

**Scheme 1:**
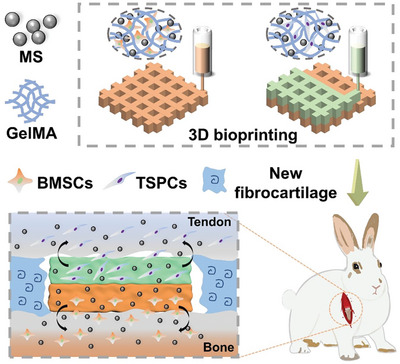
Schematic diagram of the fabrication of the biomimetic multicellular scaffolds and their application for tendon‐to‐bone interface reconstruction. The biomimetic multicellular scaffolds with structure and composition similar to the tendon‐to‐bone interface were prepared by printing tendon stem/progenitor cells (TSPCs) and bone marrow mesenchymal stem cells (BMSCs) together with bi‐layered spatial distribution. Meanwhile, the Mo‐containing silicate (MS) bioceramics were incorporated into the bioinks to endow the scaffolds with bi‐directional bioactivities. When transplanted to the rabbits rotator cuff tear (RCT) injuries, the biomimetic multicellular scaffolds could promote the integrated regeneration of tendon‐to‐bone interface.

**Figure 2:**
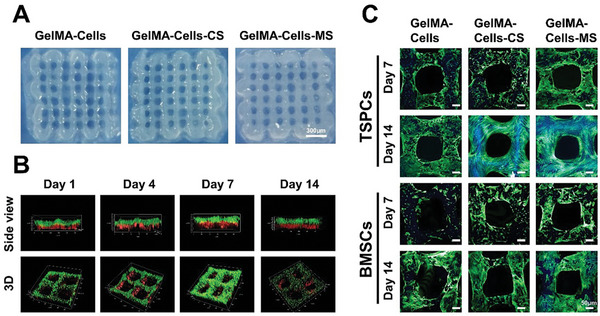
Characterization of biomimetic multicellular scaffolds containing both TSPCs and BMSCs. A) Optical photographs of biomimetic multicellular scaffolds. B) Confocal laser scanning microscopy (CLSM) images of the spatial distribution of TSPCs and BMSCs within the scaffolds after cultured for 1, 4, 7, and 14 days. TSPCs were labeled with green fluorescence in the upper layer, and BMSCs were labeled with red fluorescence in the bottom layer. C) Images of the morphology of TSPCs and BMSCs in the scaffolds. The cytoskeleton was stained in green and the nucleus was stained in blue. The spatial distribution of TSPCs and BMSCs in biomimetic multicellular scaffolds simulated the cellular composition and hierarchical structure of tendon‐to‐bone interfaces. The formation of actin filaments networks indicated that MS bioceramics were beneficial to the migration and diffusion of TSPCs and BMSCs.

In order to mimic the hierarchical structure and major cellular composition (tendon and bone) of tendon‐to‐bone interfaces, biomimetic multicellular scaffolds were designed as a bilayered structure containing TSPCs and BMSCs via dual‐channel 3D bioprinting technique. Specifically, TSPCs were distributed in the upper layers and BMSCs in the bottom layers (**Scheme** **1**). In order to thoroughly investigate the regulation effect of MS bioceramics in the multicellular system, bi‐layered scaffolds were printed with MS‐containing bioinks (GelMA‐Cells‐MS), CaSiO_3_ (CS)‐containing bioinks (GelMA‐Cells‐CS) and GelMA bioinks (GelMA‐Cells), respectively. The printed biomimetic multicellular scaffolds are shown in **Figure** **2**A. Optical images showed that the addition of CS and MS bioceramics had no effect on the shear‐thinning performance of GelMA and improved the printability of GelMA (Figure S4A–C, Supporting Information). Besides, inductively coupled plasma atomic emission spectrometry (ICP‐AES) revealed that GelMA‐Cells‐MS sustainably released Mo ions during 14 days of culture. Meanwhile, GelMA‐Cells‐CS and GelMA‐Cells‐MS exhibited nearly identical release profiles of Si and Ca ions (Figure S4D, Supporting Information).

The confocal laser scanning microscope (CLSM) images demonstrated that TSPCs (green fluorescence) were evenly distributed in the upper layers of scaffolds, and BMSCs (red fluorescence) were distributed in the bottom layers (Figure 2B). Moreover, this layered structure remained stable for 14 days. This result confirmed that the distribution of the two types of cells could be precisely controlled by using 3D bioprinting technology. In addition, the live/dead assay indicated that both BMSCs and TSPCs were uniformly distributed within the scaffolds and maintained high viability in all groups (Figure S5A,B, Supporting Information). After that, DAPI/F‐actin staining was applied to observe the morphology of BMSCs and TSPCs. Both of BMSCs and TSPCs spread well on the surface of the scaffolds after 7 days of culture. With the migration of cells and degradation of scaffolds, much more BMSCs and TSPCs were located on the surface of the scaffolds, forming actin filaments networks on day 14 (Figure 2C). Besides, after culturing for 14 days, the dense actin filaments networks formed by TSPCs and BMSCs could be observed in GelMA‐Cells‐CS and GelMA‐Cells‐MS, indicating that a large number of TSPCs and BMSCs migrated to the surface of GelMA‐Cells‐CS and GelMA‐Cells‐MS scaffolds. However, the actin filaments networks in GelMA‐Cells were clearly much sparser than these in other groups. Subsequently, a semi‐quantitative statistical analysis of the fluorescence intensity was performed. As shown in Figure S6A,B, Supporting Information, there were no significant differences in mean fluorescence intensity among these groups. Nevertheless, the fraction of actin filaments networks area in the GelMA‐Cells‐CS and GelMA‐Cells‐MS was higher than that in the GelMA‐Cells scaffolds, indicating that MS and CS bioceramics were beneficial to the migration and diffusion of encapsulated cells (Figure S6C,D, Supporting Information). Hence, the biomimetic multicellular scaffolds with stable bi‐layered structures and high cell activities were successfully fabricated.

### 2.3 Tenogenic and Osteogenic Differentiation of Biomimetic Multicellular Scaffolds

**Figure 3:**
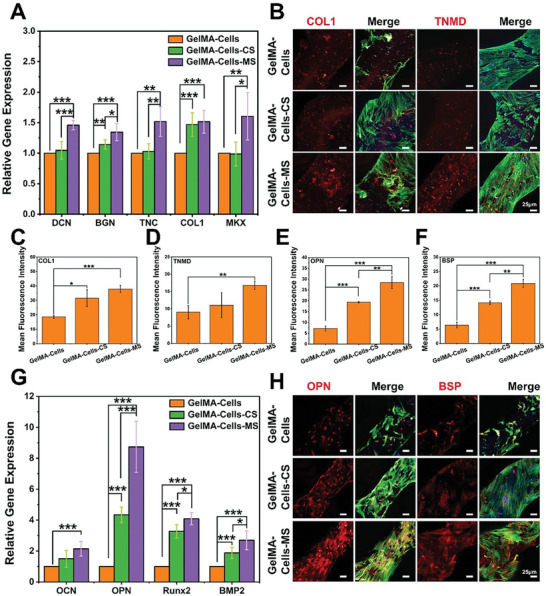
Tenogenic differentiation activity of TSPCs and osteogenic differentiation activity of BMSCs within the biomimetic multicellular scaffolds. A) Tenogenic differentiation‐related genes (DCN, TNC, BGN, COL1, and MKX) expression of TSPCs inside biomimetic multicellular scaffolds after 14 days of culture (*n* = 5). B) Immunofluorescent protein staining images of tenogenic markers (COL1 and TNMD) of TSPCs inside biomimetic multicellular scaffolds after 14 days of culture. Semi‐quantitative analysis of C) COL1, D) TNMD, E) OPN, and F) BSP expression (*n* = 3). G) Osteogenic differentiation‐related genes (OCN, OPN, Runx2, and BMP2) expression of BMSCs inside biomimetic multicellular scaffolds after 14 days of culture (*n* = 5). H) Immunofluorescent protein staining images of osteogenic markers (BSP and OPN) of BMSCs inside biomimetic multicellular scaffolds after 14 days of culture. *p < 0.05, **p < 0.01, ***p < 0.001. With the incorporation of MS particles, GelMA‐Cells‐MS exhibited the optimal capacities of simultaneously inducing tenogenic differentiation of TSPCs and osteogenic differentiation of BMSCs.

In order to investigate the specific differentiation of TSPCs and BMSCs within the 3D‐bioprinted biomimetic multicellular scaffolds, real‐time quantitative polymerase chain reaction (q‐PCR) and immunofluorescent protein staining were performed. First, as shown in **Figure** **3**A, TSPCs in GelMA‐Cells‐MS scaffolds expressed more tenogenic differentiation‐related genes (DCN, TNC, and BGN) compared with TSPCs in GelMA‐Cells‐CS and GelMA‐Cells scaffolds. In addition, the MKX expression of TSPCs in GelMA‐Cells‐MS was significantly upregulated compared to other groups. Besides, although GelMA‐Cells‐CS and GelMA‐Cells groups showed similar expression of DCN, TNC, and MKX, the expression level of COL1 in the GelMA‐Cells‐CS group was significantly higher than that in GelMA‐Cells group. Subsequently, the protein expression level of TNMD and COL1 was analyzed by immunofluorescence staining assay. Figure 3B showed that much more COL1 and TNMD proteins were expressed in GelMA‐Cells‐MS groups as compared to other groups. The semi‐quantitative analysis also confirmed that the GelMA‐Cells‐MS group had the highest expression level of TNMD and COL1 (Figure 3C,D). In addition, TSPCs in GelMA‐Cells‐CS scaffolds exhibited higher expression of COL1 than those in GelMA‐Cells scaffolds.

In addition, the expression of genes related to osteogenesis (OCN, OPN, Runx2, and BMP2) of BMSCs was also detected by q‐PCR (Figure 3G). Among these four genes, BMP2 is a powerful cytokine that regulates the function of transcription factors through activating Smad signaling during bone and cartilage development.^[^ ^22^ ^]^ Besides, OCN, a bone‐derived protein produced by osteoblasts, is highly expressed in bones and closely related to bone metabolism.^[^ ^23^ ^]^ Moreover, Runx2 can regulate osteogenic differentiation of BMSCs and its deficiency will result in the lack of mature osteoblasts and incomplete bone formation.^[^ ^24^ ^]^ OPN, an abundant bone extracellular matrix protein, plays an important role in mineralization, cell adhesion, and cell migration.^[^ ^25^ ^]^ All of their expression in the GelMA‐Cells‐MS group was higher than in GelMA‐Cells. In particular, the expression of BMP2, OPN, and Runx2 was markedly upregulated in GelMA‐Cells‐MS compared to GelMA‐Cells‐CS. Moreover, the expression of OPN, Runx2, and BMP2 in the GelMA‐Cells‐CS group was much higher than that in the GelMA‐Cells group. Immunofluorescence staining assay and semi‐quantitative analysis indicated that osteogenesis‐related protein (OPN and BSP) expression of BMSCs in GelMA‐Cells‐MS was higher than that in other groups (Figure 3E,F,H). Meanwhile, their expression was also upregulated in the GelMA‐Cells‐CS group as compared to GelMA‐Cells.

Encouragingly, MS bioceramics possessed the capacity of simultaneously inducing tenogenic differentiation of TSPCs and osteogenic differentiation of BMSCs. Meanwhile, compared with GelMA‐Cells, GelMA‐Cells‐CS also displayed the ability to promote osteogenic differentiation of BMSCs and the COL1 expression of TSPCs. It was reported that inorganic ions could build a bioactive microenvironment to stimulate specific differentiation of multiple cells.^[^ ^26^ ^]^ Therefore, it could be inferred that Mo, Ca, and Si ions released by the silicate bioceramics activated the cells and induced them toward specific differentiation. Notably, it has been proven that Ca and Si ions could increase osteogenic differentiation of HBMSCs by activating BMP‐2 signaling pathway.^[^ ^27^ ^]^ Moreover, Si ions were able to stimulate the synthesis of COL1 through the BMP‐2/Smad/RUNX2 signaling pathway.^[^ ^28^ ^]^ Interestingly, MS bioceramics exhibited more significant effects on the differentiation of TSPCs and BMSCs than CS, indicating that Mo ions played indispensable roles in enhancing the bi‐directional function. In particular, MS‐containing scaffolds significantly upregulated the tenogenic markers, DCN, TNC, and TNMD, while CS‐containing scaffolds showed no apparent effects on their expression. Moreover, compared to CS bioceramics, MS bioceramics exhibited significantly higher impact on the expression of osteogenic markers, OPN, BSP, and Runx2, in BMSCs. Hence, it is reasonable to speculate that Mo ions enhanced the bi‐directional bioactivities of silicate bioceramics. To verify this speculation, the effects of Mo ions on the activities of TSPCs and BMSCs were further explored.

### 2.4 Effect of Mo Ions on Proliferation, Migration, and Differentiation of TSPCs and BMSCs

In order to figure out the effects of Mo ions on the activities of TSPCs and BMSCs and the relevant mechanism, the culture medium with a gradient concentration of Mo ions was used to incubate the cells. As mentioned above, ionic release profiles revealed that the concentration of Mo ions released by GelMA‐Cells‐MS scaffolds was maintained below 10 mg L^−1^ during the whole culture period. Hence, TSPCs and BMSCs were cultured with the culture medium supplemented with Mo ions in the range of 0–10 mg L^−1^ (2, 5, and 10 mg L^−1^).

**Figure 4:**
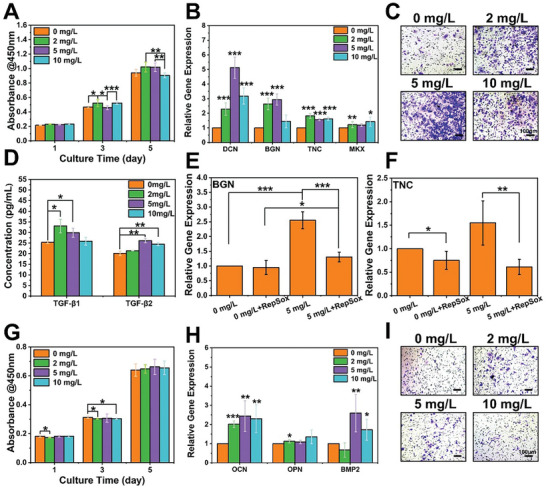
Effect of Mo ions on the proliferation, migration, and differentiation of both TSPCs and BMSCs. A) Effect of different concentrations of Mo ions on the proliferation activity of TSPCs during 5 days of culture (*n* = 6). B) The expression level of tenogenic differentiation‐related genes (DCN, BGN, TNC, MKX) of TSPCs after culturing with different concentrations of Mo ions (*n* = 5). C) Migration of TSPCs induced with different concentrations of Mo ions. D) The concentration of TGF‐*β*1 and TGF‐*β*2 secreted by TSPCs after cultured with different concentrations of Mo ions. Relative expression level of E) BGN and F) TNC of TSPCs after cultured with 0 and 5 mg L^−1^ of Mo ions under TGF‐*β* inhibitor (RepSox) treatment. G) Effect of different concentrations of Mo ions on the proliferation activity of BMSCs during 5 days of culture (*n* = 6). H) The expression level of osteogenic differentiation‐related genes in BMSCs after cultured with different concentrations of Mo ions (*n* = 5). I) Migration of BMSCs induced with different concentrations of Mo ions. *p < 0.05, **p < 0.01, ***p < 0.001. Mo ions could stimulate osteogenic differentiation of BMSCs and tenogenic differentiation of TSPCs via activating TGF‐*β* pathway, as well as promote the migration activity of both TSPCs and BMSCs.

First, the effects of Mo ions on the proliferation, migration, and differentiation activities of TSPCs were investigated. **Figure** **4**A showed that the addition of Mo ions had negligible influences on the proliferation of TSPCs within 5 days. Nevertheless, it could be found that 2 and 5 mg L^−1^ of Mo ions significantly improved the expression level of tenogenic differentiation‐related genes (DCN, BGN, and TNC) in TSPCs. Moreover, TSPCs cultured with 2 and 10 mg L^−1^ Mo ions expressed more MKX compared to 0 mg L^−1^ Mo ions (Figure 4B). Besides, the effect of Mo ions on TSPCs migration was explored by transwell assay. As shown in Figure 4C and Figure S7A, Supporting Information, the migration of TSPCs was significantly promoted by Mo ions treatment. To further explore the mechanism related to Mo ions inducing tenogenic differentiation of TSPCs, transforming growth factor beta (TGF‐*β*) secreted by TSPCs was tested. TGF‐*β* is an inducer for tendon growth. Previous studies found that disruption of TGF‐*β* signaling resulted in the loss of most tendons and ligaments in the extremities, trunk, tail, and head.^[^ ^29^ ^]^ Therefore, the expression of TGF‐*β*1 and TGF‐*β*2 was analyzed to confirm whether Mo ions promoted TSPCs tenogenic differentiation via the TGF‐*β* pathway. Figure 4D confirmed that Mo ions enhanced the expressions of TGF‐*β*1 and TGF‐*β*2. In addition, after treating with a pharmacologic inhibitor of TGF‐*β* signaling, TSPCs obviously expressed less tenogenic differentiation‐related genes (BGN and TNC) (Figure 4E,F). Thus, it could be inferred that Mo ions stimulated tenogenic differentiation of TSPCs through TGF‐*β* pathways.

Then, the effects of Mo ions on the proliferation, migration, and differentiation activities of BMSCs were investigated. Figure 4G showed that the addition of Mo ions had negligible influences on the proliferation of BMSCs within 5 days. As shown in Figure 4H, 2 mg L^−1^ of Mo ions were able to significantly increase the expression of osteogenic genes, OCN, and OPN. Meanwhile, 5 and 10 mg L^−1^ of Mo ions upregulated the gene expression of BMP2 and OPN. Afterward, the effect of Mo ions on the migration activity of BMSCs was investigated. The crystal violet staining images and their statistical results confirmed that the migration activity of BMSCs could be obviously enhanced by Mo ions treatment (Figure 4I and Figure S7B, Supporting Information). Based on the above results, 2–10 mg L^−1^ of Mo ions could activate TSPCs and BMSCs and induce them toward specific differentiation. On the one hand, some biomaterials containing Mo element have been applied to construct tendon tissue scaffolds,^[^ ^30^ ^]^ but there are few studies that clearly reveal the positive effects of Mo ions on the tendon regeneration to our knowledge. In this work, Mo ions were testified to promote tenogenic differentiation of TSPCs via the TGF‐*β* pathway. TGF‐*β* was demonstrated to play important roles in regulating tendon repair.^[^ ^29^, ^31^ ^]^ Previous studies found that TGF‐*β* could increase the activities of TSCPs through activation of TGF‐*β*/Smad‐3 signaling pathway and MAPK pathways.^[^ ^31^ ^]^ On the other hand, Mo ions were found to upregulate the osteogenic differentiation of BMSCs. As previously reported, Mo ions could induce the osteogenesis by enhancing ALP expression and calcium nodules, as well as regulating the differentiation direction of BMSCs through the HIF‐1*α* signaling pathway.^[^ ^13^ ^]^ Moreover, Mo ions could mediate the bone formation and resorption balance to facilitate bone repair by regulating the mitochondrial biogenesis.^[^ ^32^ ^]^ Hence, Mo ions might stimulate the osteogenic differentiation of BMSCs by HIF‐1*α* signaling pathway and promote the tenogenic differentiation of TSPCs through TGF‐*β*‐related signaling, thus endowing Mo ions with the dual bioactivity. Taken together, Mo ions with moderate concentration promoted the specific differentiation of both TSPCs and BMSCs. Thus, it could be inferred that Mo ions enhanced the dual bioactivities of silicate bioceramics and played vital roles in bi‐directional differentiation function of the multicellular scaffolds.

### 2.5 RCT Regeneration In Vivo

In this study, a RCT model of rabbits was established to evaluate the repair effect of the biomimetic multicellular scaffolds in vivo (Figure S8, Supporting Information). Rabbits were randomized into four groups: Blank, GelMA‐Cells, GelMA‐MS, and GelMA‐Cells‐MS. After 12 weeks postoperatively, rabbits were sacrificed and the rotator cuff tissues were collected for subsequent analysis.

**Figure 5:**
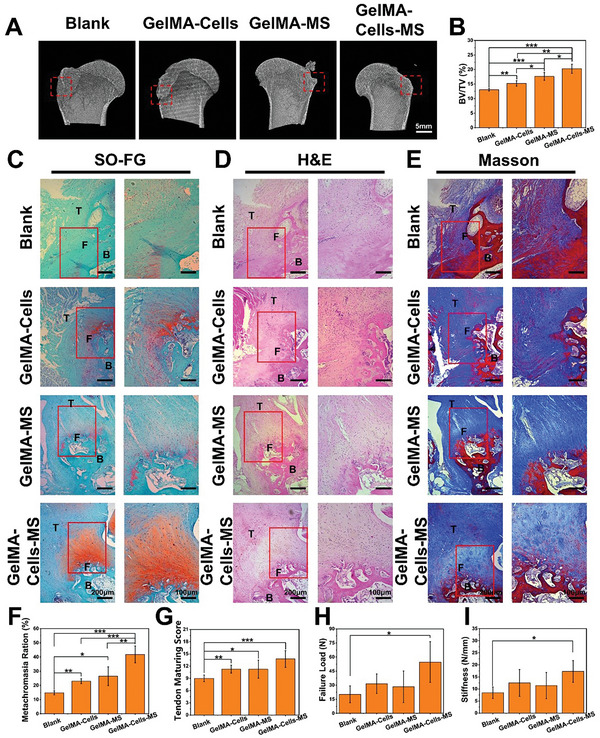
Biomimetic multicellular scaffolds promote rabbit rotator cuff tear (RCT) repair in vivo. A) Representative micro‐CT images of the humeral head at 12 weeks postoperatively. B) The bone volume fraction (BV/TV) values of different groups at 12 weeks (*n* = 4). C) Representative Safranine O‐Fast Green (SO‐FG) staining images of the regenerated fibrocartilage after 12 weeks of post‐surgery (T: tendon, F: fibrocartilage, B: bone). D) Representative hematoxylin eosin (H&E) staining images of tendon‐to‐bone interfaces after 12 weeks of post‐surgery. E) Representative Masson staining images of tendon‐to‐bone interfaces after 12 weeks of post‐surgery. F) The metachromasia ratio of different groups at 12 weeks (*n* = 4). G) Tendon maturing scores of different groups (*n* = 4). *p < 0.05, **p < 0.01, ***p < 0.001. The H) failure load and I) stiffness of the tendon at the interface site after 12 weeks of post‐surgery (*n* = 4). *p < 0.05, **p < 0.01, ***p < 0.001. 3D bioprinted GelMA‐Cells‐MS scaffolds exhibited the best capacities of promoting bone repair, tendon maturation, fibrocartilage formation, collagen deposition, and biomechanical improvement, leading to the satisfactory regeneration of tendon‐to‐bone interfaces in vivo.

First, 3D Micro‐CT was used to reconstruct the humeral defects in RCT. As shown in **Figure** **5**A, the bone tissues in the Blank and GelMA‐Cells groups were discontinuous. In contrast, intact cortical bone formation could be observed in GelMA‐MS and GelMA‐Cells‐MS groups. Quantitative analysis results further confirmed that GelMA‐Cells‐MS groups had the highest bone volume/total volume (BV/TV) values, indicating that GelMA‐Cells‐MS groups possessed the best bone regeneration ability compared with other groups (Figure 5B). Then, histological analysis of the rotator cuff tissues was conducted by continuous sections. Due to the regeneration of fibrocartilage being crucial for the repair of rotator cuff injuries, Safranine O‐Fast Green (SO‐FG) staining was applied to evaluate the neo‐fibrocartilage formation in tendon‐to‐bone interfaces. Figure 5C,F showed that GelMA‐Cells‐MS groups had the highest metachromasia ratio at the tendon‐to‐bone interfaces as compared with other groups. Besides, more ordered organizations in GelMA‐MS and GelMA‐Cells‐MS groups were observed through hematoxylin‐eosin staining (H&E) (Figure 5D). As shown in Figure S9, Supporting Information, a large amount of fatty infiltration, which was regarded as a sign of degenerative changes in tendon diseases, was observed in Blank and GelMA‐Cells groups but was hardly found in GelMA‐MS and GelMA‐Cells‐MS groups. What's more, the maturity level of the regenerated tendon tissues was evaluated based on the H&E staining images. GelMA‐Cells‐MS groups exhibited the highest tendon maturity level according to the tendon‐maturing scoring standard (Figure 5G). In addition, Masson staining results demonstrated that the regenerated tissues in scaffolds‐implanted groups exhibited better organization and more collagen deposition than those in Blank groups (Figure 5E). Besides, according to the results of biomechanical test, the failure load and stiffness of the tendon at the interface site in GelMA‐Cells‐MS groups were both higher than other groups, indicating the rotator cuffs repaired by GelMA‐Cells‐MS exhibited better mechanical performance (Figure 5H,I).

Therefore, it could be found that the biomimetic multicellular scaffolds promoted new bone formation, tendon maturation, junctional fibrocartilage formation, collagen deposition, and the improvement of mechanical performance, thus accelerating the integrated repair of tendon‐to‐bone injury. These satisfying results could be mainly attributed to the following reasons:

1) To regenerate the complex soft‐to‐hard tissue interfaces, the synchronous repair of multiple tissues is required.^[^ ^33^ ^]^ Biomimetic strategy is considered as a feasible method for the integrated regeneration of complex tissues.^[^ ^4^, ^34^ ^]^ Currently, some studies have prepared scaffolds that only imitate the physical structure of tendon‐to‐bone,^[^ ^5^, ^33^ ^]^ but there are still limitations in inducing multiple tissues regeneration due to the huge differences between these bionic scaffolds and natural tendon‐to‐bone tissues. In contrast, the biomimetic multicellular scaffolds have a hierarchical structure and multicellular composition similar to tendon‐to‐bone, which reproduces the physical and biological features of tendon‐to‐bone as closely as possible. Taking advantage of these multi‐level biomimetic features, the biomimetic multicellular scaffolds can better participate in the natural repair process, which leads to the orderly arrangement of multiple cells and extracellular matrices at the interface to accelerate the regeneration of tendon‐to‐bone.
2) Hypocellularity is one of the main reasons for the difficulty in restoring the injured tendon.^[^ ^35^ ^]^ In recent years, delivering exogenous cells has been a feasible approach to solving this issue.^[^ ^36^ ^]^ The researchers found that cell sheets loaded with TSPCs could enhance tendon toughness by providing exogenous TSPCs.^[^ ^37^ ^]^ At the same time, supplying BMSCs to the bone defects is also an effective way to resolve the bone defects.^[^ ^38^ ^]^ As a result, the multicellular scaffolds prepared by bioprinting can accurately deliver BMSCs and TSPCs in a controllable manner, which has great potential for relieving hypocellularity in tendon‐to‐bone defects.
3) By releasing multiple bioactive ions, especially Mo ions, the Mo‐containing bioinks build a beneficial microenvironment for the integrated regeneration of tendon‐to‐bone interfaces. Compared with the strategy of pre‐differentiation and extracellular matrices,^[^ ^5^, ^39^ ^]^ our strategy of designing the composite bioinks containing MS bioceramics showed the clear advantages of inducing the stable and sustainable bi‐lineage differentiation of multiple cells. Interestingly, Mo ions can enhance mitochondrial function and cellular metabolism, which is crucial in tissue regeneration.^[^ ^13^, ^14^, ^32^ ^]^ Therefore, Mo ions can serve as functional cellular modulators to activate multiple cells, thus facilitating the integrated regeneration of bone, tendon, and their junctional fibrocartilage.

## 3 Conclusion

In conclusion, we successfully prepared a biomimetic multicellular scaffold via 3D bioprinting for the regeneration of tendon‐to‐bone. First, by the bilayered spatial distribution of biomimetic inks containing TSPCs/BMSCs and Mo‐containing silicate (MS) bioceramics, the bionic simulation of structure and cellular components of tendon‐to‐bone interfaces was realized. Second, using bioactive ions released by MS bioceramics, especially Mo ions, the biomimetic scaffolds exhibited the bi‐directional bioactivity of tenogenic and osteogenic differentiation, which was required for the reconstruction of tendon‐to‐bone interfaces. Furthermore, biomimetic multicellular scaffolds possessed the excellent capacity to induce integrated regeneration of tendon‐to‐bone interfaces in vivo. Our study showed that 3D multicellular bioprinting of biomimetic inks containing inorganic bioceramics presented a promising approach for the regeneration of soft‐to‐hard tissue interfaces.

## 4 Experimental Section

### Fabrication and Characterization of Mo‐Containing Silicate Bioceramics

Mo‐containing silicate bioceramics were synthesized through a chemical coprecipitation method by using calcium nitrate tetrahydrate (Ca (NO_3_)_2_·4H_2_O), sodium metasilicate nonahydrate (Na_2_SiO_3_·9H_2_O) and ammonium molybdate tetrahydrate (H_24_Mo_7_N_6_O_24_·4H_2_O) as raw materials. All raw materials were purchased from Sinopharm Chemical Reagent Co., Ltd, Shanghai, China. Briefly, 59.04 g of Ca (NO_3_)_2_·4H_2_O was dissolved in 500 mL of deionized water. Subsequently, H_24_Mo_7_N_6_O_24_·4H_2_O was added into Ca (NO_3_)_2_·4H_2_O solution (molar ratio: Mo/Ca = 0%, 2%, 5%, and 10%) to obtain Ca‐Mo solution. Then, 71.05 g of Na_2_SiO_3_·9H_2_O was added to 500 mL of deionized water to get silicate solution. Next, the Ca‐Mo solution was added to the silicate solution dropwise. After vigorously stirring for 24 h at ambient temperature, the mixture was filtered to get the precipitates. The precipitates were washed with deionized water and ethanol three times, subsequently dried at 60 °C for 24 h, calcined at 800 °C for 3 h, and ground to obtain Mo‐containing silicate bioceramics. Four kinds of Mo‐containing silicate bioceramics were denoted according to their molar ratio of Mo/Ca: CS (0%), 2Mo‐CS (2%), 5Mo‐CS (5%), 10Mo‐CS (10%).

The phase of Mo‐containing silicate bioceramics was characterized by an X‐ray diffractometer (XRD, Geigerflex, Rigaku Co, Japan). Then, the morphology and elemental distribution of 10Mo‐CS (MS) were observed by SEM (SU8220, HITACHI, Tokyo, Japan) equipped with EDS.

### Cell Culture

BMSCs were isolated from rabbit femur tissues. TSPCs were extracted from rabbit tendon tissues. Both of BMSCs and TSPCs were cultured in Minimum Essential Medium‐alpha (MEM‐*α*, Gibco, USA) medium supplemented with 10% fetal bovine serum (FBS, Gibco, USA) and 1% penicillin‐streptomycin (P/S, Gibco, USA) under 37 °C and 5% CO_2_ incubator.

### Preparation of Bioinks with Different Compositions

Gelatin methacryloyl (GelMA) was synthesized according to the previous report.^[^ ^16^ ^]^ In order to obtain a 12% GelMA solution, 0.05 g of lithium phenyl‐2,4,6‐trimethylbenzoylphosphinate (LAP) and 1.2 g of freeze‐dried GelMA foam were added into 10 mL phosphate‐buffered saline (PBS) and dissolved at 60 °C. All the following steps were performed on a laminar flow cabinet (ESCO, Class II Biological Safety Cabinet). The obtained 12% GelMA solution was sterilized through 0.22 µm filters. Ultraviolet light was utilized to sterilize MS bioceramics for 4 h. Then, 1 mL cell suspensions (TSPCs or BMSCs) (3x10^6^ cells mL^−1^) and 1 mL MS solution (the mass ratio of MS to GelMA was 0%, 1.5%, 3%, and 4.5%) were mixed with 2 mL of 12% GelMA solution to obtain the bioinks with different MS contents. Finally, the prepared bioinks were denoted as GelMA, GelMA‐1.5MS, GelMA‐3MS, and GelMA‐4.5MS, respectively.

### Rheology Test of the Bioinks

The rheological properties of bioinks were determined by rheometer (MCR301, Anton‐Paar, Austria). The viscosities of bioinks were tested at 20 °C with shear rate range of 0.1–1000 s^−1^.

### Evaluation of the Printability of the Bioinks

Stereomicroscopes (S6D, Leica, Germany) were used to observe the grid‐like structures of the 3D‐printed scaffolds. Printability was calculated by Image‐J software according to the equation: Pr = L^2^/(16A). Pr was printability, L was the perimeter of the square hole, and A was the area of the square hole.

### 4.1 Bioprinting of TSPCs/BMSCs‐Laden Scaffolds with Different MS Contents

A multi‐channel 3D bioprinting platform (Bioscaffolder 3.2, Gesim, German) was used to fabricate 3D bioprinted scaffolds. The whole printing process was carried out on a laminar flow cabinet (ESCO, Class II Biological Safety Cabinet) after sterilized by ultraviolet light and 75% alcohol. First, bioinks were added into stainless syringes and stored at 4 °C for 20 min. Then, the scaffolds were prepared by extruding the bioinks with pressure between 20 and 30 kPa at 20.5 °C, and crosslinked by blue light (the wavelength was 405 nm) for 15 s. Finally, 3D bioprinted cell‐laden scaffolds were cultured in 24‐well plates with 1 mL MEM‐*α* culture medium and incubated in a humidified incubator with 5% CO_2_ at 37 °C. The medium was changed every 3 days.

### Live/Dead Staining Assay

Cell viability was analyzed by Calcein‐AM/PI Double Staining Kit (Dojindo, Japan). Briefly, each scaffold in 24‐well plates was soaked in 1 mL mixture solution (AM:PI:PBS = 2:1:1000) in dark conditions for 20 min. The live cells with green fluorescence excited by 488 nm laser and dead cells with red fluorescence excited by 556 nm laser were observed by a fluorescence microscope (DMi8 S, Leica, Germany).

### 4.2 Bioprinting of Biomimetic Multicellular Scaffolds

Biomimetic multicellular scaffolds were constructed by using a dual‐channel printing method. First, BMSCs‐laden bioinks and TSPCs‐laden bioinks were transferred into two stainless syringes, respectively. Then, the two syringes were mounted in the two channels of the bioprinter. The printing steps and conditions were the same as mentioned above. Specifically, TSPCs‐laden bioinks were first printed for four layers and then BMSCs‐laden bioinks were printed for four layers to form the biomimetic multicellular scaffolds. The scaffolds prepared by different bioinks were recorded as GelMA‐Cells (scaffolds with cells), GelMA‐Cells‐CS (scaffolds with cells and CaSiO_3_), and GelMA‐Cells‐MS (scaffolds with cells and MS).

### Ionic Release

In order to measure the ionic release behaviors of the scaffolds, the scaffolds were cultured in 1 mL of culture medium at 37 °C. Then the medium was collected after culturing for 1, 2, 3, 5, 7, 10, and 14 days. The concentration of Mo and Si elements in the culture medium was tested by inductively coupled plasma atomic emission spectroscopy (ICP‐AES, 715‐ES, Varian, USA).

### Spatial Distribution of Cells within the Scaffolds

First, BMSCs and TSPCs were labeled with fluorescent markers (CellTracker, Invitrogen, USA) before encapsulating into bioinks. TSPCs were marked with green fluorescence and BMSCs were marked with red fluorescence. Subsequently, the whole printing and culture processes were carried out under dark conditions. Then, the biomimetic multicellular scaffolds were fixed with 4% paraformaldehyde at different time points (days 1, 4, 7, 14). The spatial distribution of BMSCs and TSPCs in the biomimetic scaffolds was observed by confocal laser scanning microscopy (CLSM, TCS SP8, Leica, Germany).

### Cell Morphology Assay

Diamidinophenylindole (DAPI, Sigma‐Aldrich) and Alex Fluor 488‐conjugated phalloidin (Molecular Probes, USA) were used to observe the morphology of cells encapsulated in the scaffolds. First, the scaffolds were fixed with 4% paraformaldehyde for 30 min and then permeabilized with 0.5% Triton‐X solution for 5 min. Next, the scaffolds were stained by DAPI for 10 min and phalloidin for 45 min, respectively. Each step was washed with PBS three times for 5 min. Finally, the cells were observed by confocal laser scanning microscopy (TCS SP8, Leica, Germany).

### Gene Expression of Cells within the Scaffolds

Gene expression level of cells within the biomimetic scaffolds was analyzed by RT‐qPCR. In order to facilitate the RNA extraction of the two types of cells, the upper layers with TSPCs and the bottom layers with BMSCs were printed separately and then put together for co‐culture. After 14 days of co‐culture, scaffolds were digested with GelMA Lysis Buffer (EFL, EFL‐GM‐LS‐001, Suzhou, China) for 2 h and then centrifugated at 1000 rpm for 5 min to collect cells. Then, the cells were treated with Tirzol (TAKARA, Japan) reagent. After that, RNA was extracted by mixing Tirzol reagent with trichloromethane (LINGFENG, Shanghai) and isopropanol (LINGFENG, Shanghai), respectively. Subsequently, cDNA was obtained by using PrimeScript first Strand cDNA synthesis kit (TOYOBO, Japan) according to the manufacturer's instructions. In the end, the RT‐qPCR process was performed by StepOnePlus Real‐time systems (Applied Biosystems, USA). Gene expressions were calculated by the 2^−ΔΔCT^ method. The primer sequences of the related genes were listed in Table S3, Supporting Information.

### Protein Expression of BMSCs and TSPCs within the Scaffolds

The specific protein expression of TSPCs and BMSCs within the scaffolds was detected by immunofluorescence staining assay. Briefly, in order to facilitate the immunofluorescence characterization of BMSCs and TSPCs, the scaffolds were printed separately as described above. First, the scaffolds were fixed in 4% paraformaldehyde for 40 min after 14 days of culture. Then, the scaffolds were permeabilized with 0.1% Triton‐X100 for 5 min and washed with PBS for three times. Next, 5% Bovine Serum Albumin (BSA) was added to block the scaffolds at ambient temperature for 30 min, and subsequently primary antibody was added to incubate the scaffolds overnight at 4 °C. After removing the primary antibody, the second antibody was added and incubated at 37 °C for 1 h in the dark environment. Finally, the immunofluorescence staining images of TSPCs and BMSCs were obtained by confocal laser scanning microscopy (TCS SP8, Leica, Germany). Semi‐quantitative analysis of the protein expression level was conducted by Image‐J software.

### Effect of Mo Ions on the Proliferation of TSPCs and BMSCs

CCK‐8 (Cell counting Kit‐8, Dojindo, Japan) assay was used to determine the effect of Mo ions on the proliferation activity of TSPCs and BMSCs. First, cells were seeded on 96‐well plates (1000 cells well^−1^) and incubated with culture medium containing different concentrations of Mo ions (0, 2, 5, 10 mg L^−1^) for 1, 3, and 5 days. At each time point, 100 µL culture medium containing 10% CCK‐8 was added into each well and incubated at 37 °C for 1 h. At last, a microplate reader (SpectraFluor Plus, Tecan, Germany) was utilized to test the absorbance of the medium at 450 nm.

### Effect of Mo Ions on the Specific Differentiation of TSPCs and BMSCs

RT‐qPCR was used to analyze genes expression of the cells cultured with different concentrations of Mo ions (0, 2, 5, 10 mg L^−1^). First, cells were seeded on 6‐well plates and incubated with culture medium containing Mo ions for 5 days. Then, the RNA of the cells was extracted by Tirzol (TAKARA, Japan) reagent. Subsequently, PrimeScript first Strand cDNA synthesis kit (TOYOBO, Japan) was used to obtain cDNA. Last, the RT‐qPCR process was performed by StepOnePlus Real‐time systems (Applied Biosystems, USA). The expression of genes was calculated by the 2^−ΔΔCT^ method.

### Effect of Mo Ions on the Migration of TSPCs and BMSCs

The effect of Mo ions on the migration of TSPCs and BMSCs was examined by transwell assay. 300 µL of basal medium containing 10 000 cells was added to the upper chamber, and 700 µL of basal medium containing different concentrations of Mo ions (2, 5, and 10 mg L^−1^) was added into the lower chamber. After 6 h of incubation, the cells were fixed in 4% paraformaldehyde for 20 min. Next, the cells on the upper side of the chamber were wiped off with a cotton swab. Finally, the cells migrated to the back side of the chamber were stained with crystalline violet and observed by optical microscope.

### Effect of Mo Ions on the Expression of TGF‐*β*

Growth factor TGF‐*β* secreted from TSPCs was detected by TGF‐*β* ELISA kits according to the manufacturer's protocol. In order to explore whether Mo ions induced tenogenic differentiation of TSPCs through TGF‐*β*‐related pathways, TSPCs were cultured in medium with TGF‐*β* inhibitor (RepSox, medchemexpress) (20 µm). Then, the expression of tenogenic differentiation‐related genes was determined by RT‐qPCR. The procedure of RT‐qPCR was the same as mentioned above.

### Animal Surgery

The rabbit RCT model was established to evaluate the tendon‐to‐bone interface reconstruction ability of biomimetic multicellular scaffolds in vivo. All animal experiments were performed in accordance with the guidelines approved by the Institutional Animal Care and Use Committee of Nanjing First Hospital, Nanjing Medical University (DWSY‐2102466). In this experiment, 16 male New Zealand white rabbits (3 ± 0.5 kg) were selected and randomly divided into four groups: Blank (no scaffolds), GelMA‐Cells (MS‐free scaffolds), GelMA‐MS (cell‐free scaffolds) and GelMA‐Cells‐MS (scaffolds containing MS and cells). First, the rabbits were anesthetized and fixed in the supine position. After shaving and disinfecting the skin, the skin and the deltoid muscle were incised to expose the rotator cuff tissue. Then, the supraspinatus tendon was isolated along the tibia. The supraspinatus tendon was passed through by 5 # thread and a micro drill (5 mm in diameter) was used to drill a hole at the great nodule. Afterward, the scaffolds were placed in the hole and the supraspinatus tendon was sutured to the bone. Finally, the wound was closed in turn.

### Micro‐CT Analysis

After 12 weeks of post‐surgery, the rabbits were sacrificed. The rotator cuff tissues were collected and then soaked in 4% paraformaldehyde for 24 h. The surrounding muscle tissues were stripped by scalpel blade. All samples were analyzed using Micro‐CT (Skyscan1172, Germany) to assess the new bone formation of the humerus. Subsequently, 3D images were reconstructed by the CT‐Volume software, and the bone volume/total volume (BV/TV) value was calculated by CT‐Analyzer program.

### Histological Staining

All samples were decalcified with 10% EDTA (Servicebio, Shanghai, China) for 1 month. Then, they were dehydrated and embedded in paraffin. Subsequently, the samples were sectioned into 5 µm thickness by Rotating Slicer (Leica, Germany). Next, hematoxylin eosin staining (H&E) kits (Beyotime Biotechnology, Shanghai), Safranine O‐Fast Green staining kits (Solarbio, Beijing, China) and Masson staining kits (Solarbio, Beijing, China) were applied to staining pathological sections according to the manufacturer's requirements. The staining results were observed by Optical Microscope (Leica, Germany). Quantitative analysis of the area ratio of fibrocartilage at the tendon‐to‐bone interface was calculated by Image‐J software. After that, the histological score of all groups was evaluated and the scoring criterion was shown in Table S4, Supporting Information.

### Biomechanical Test

The biomechanical test of all samples was performed by a universal mechanical testing machine (Instron‐5566) (Figure S10, Supporting Information). The excess tissues in the rotator cuff were removed and only the supraspinatus tendon‐muscle and bone complexes were kept. To be able to hold the complexes better, medical gauzes and sandpapers were sewed to the supraspinatus muscle. After applying a preload of 1 N with a test speed of 5 mm min^−1^ (strain rate of 0.8% s^−1^), all complexes underwent a total of 20 cycles of pre‐treatment. Finally, the force‐displacement curve and failure load were recorded. Based on the linear region before the maximum load in the force‐displacement curve, the stiffness was calculated.^[^ ^39^, ^40^ ^]^

### Statistical Analysis

All data were expressed as means ± standard deviation and analyzed by One‐way ANOVA using Origin 2018 software. Significant difference was considered when *p < 0.05, **p < 0.01, ***p < 0.001.

## Conflict of Interest

The authors declare no conflict of interest.

## Author Contributions

L.D. and C.W. conceived the project. L.D. prepared and characterized the materials. L.D. and H.Z. performed the in vitro experiments. L.D., H.Z., Y.W., and J.W. conducted the in vivo experiments. L.D. and F.H. collected and analyzed data. L.D., C.Q., H.Z., and J.X. wrote the manuscript. Y.X., Z.H., and C.W. supervised the project.

## Supporting information

Supporting Information

## Data Availability

The data that support the findings of this study are available from the corresponding author upon reasonable request.
