# Supplementary material for: Multicellular Bioprinting of Biomimetic Inks for Tendon‐to‐Bone Regeneration
Source: Adv Sci (Weinh). 2023 Apr 29;10(21):2301309. doi: 10.1002/advs.202301309 (PMC10375072; doi:10.1002/advs.202301309)
Supplement: Supplementary file 1 — Supporting Information [file ADVS-10-2301309-s001.pdf]

ADVANCED SCIENCE
Open Access

Supporting Information

for *Adv. Sci.*, DOI 10.1002/advs.202301309

Multicellular Bioprinting of Biomimetic Inks for Tendon-to-Bone Regeneration

Lin Du, Chen Qin, Hongjian Zhang, Fei Han, Jianmin Xue, Yufeng Wang, Jinfu Wu, Yin Xiao, Zhiguang Huan and Chengtie Wu**

Supporting Information

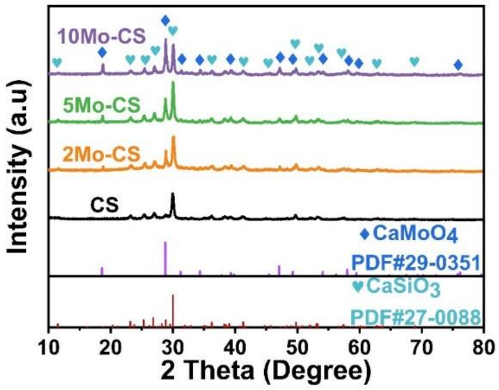

Figure S1. XRD pattern of Mo-containing silicate bioceramics with different contents of Mo (0%, 2%, 5% and 10%).

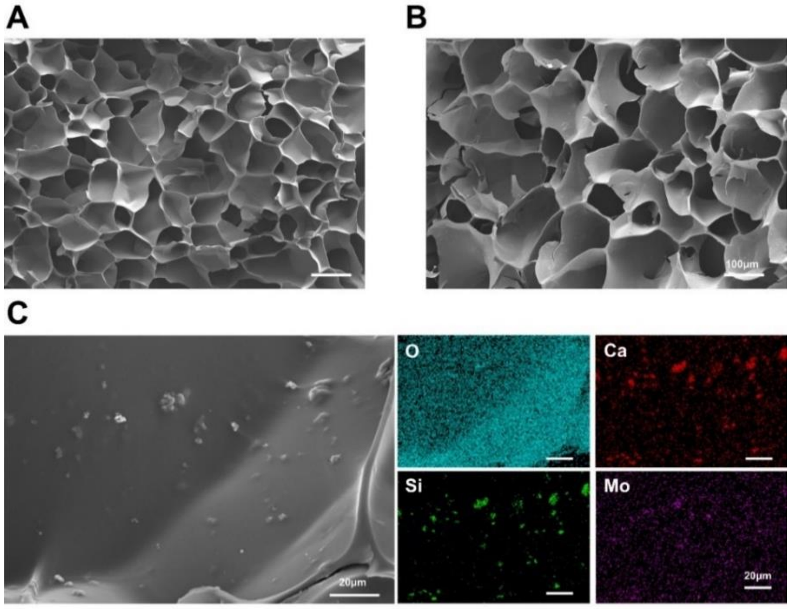

Figure S2. (A) SEM image of GelMA hydrogels. (B) SEM image of GelMA-MS hydrogels. (C) SEM images and the corresponding EDS elemental mapping of GelMA-MS hydrogels. **MS particles were uniformly distributed within the hydrogels and had neglectable effects on the macroporous structure of GelMA hydrogels.**

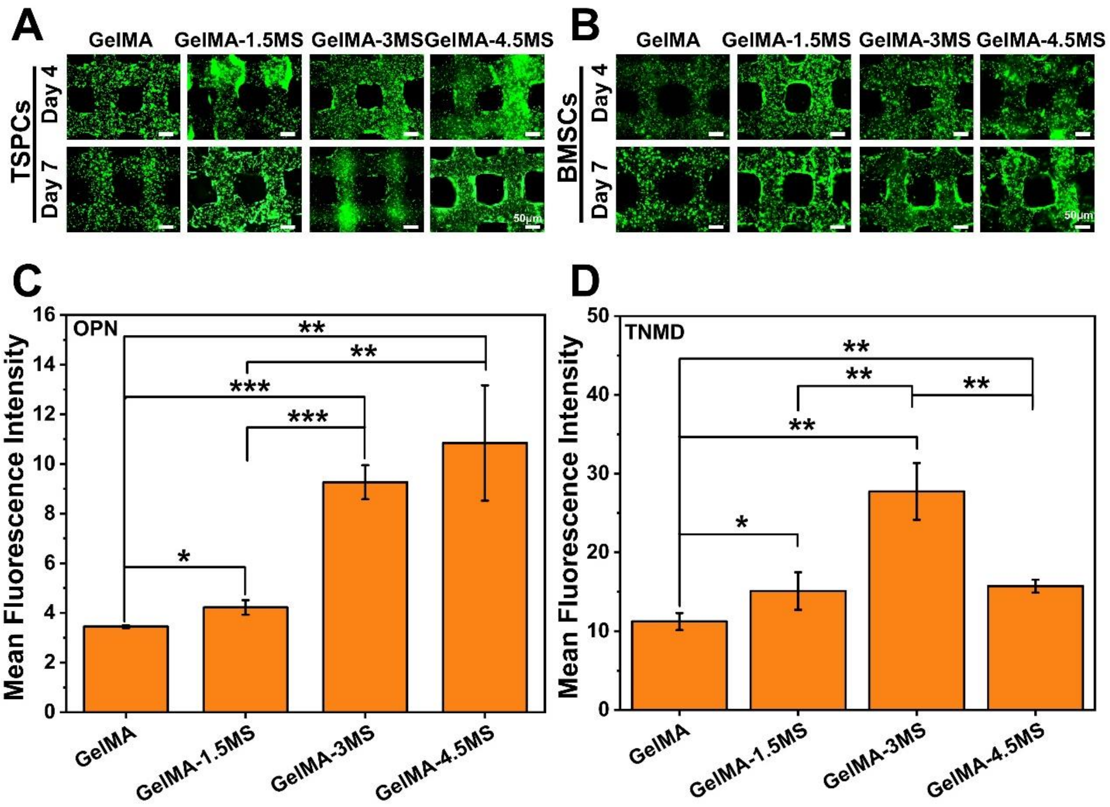

Figure S3. Live/dead assay of (A) 3D bioprinted TSPCs-laden scaffolds with different concentrations of MS bioceramics and (B) 3D bioprinted BMSCs-laden scaffolds with different concentrations of MS bioceramics. (C) Semi-quantitative analysis of OPN protein expression in 3D bioprinted BMSCs-laden scaffolds (n = 3). (D) Semi-quantitative analysis of TNMD protein expression in 3D bioprinted TSPCs-laden scaffolds (n = 3). *$p < 0.05$, **$p < 0.01$, ***$p < 0.001$. **MS-containing bioinks supported the long-term survival of TSPCs and BMSCs and improved their expression of specific markers.**

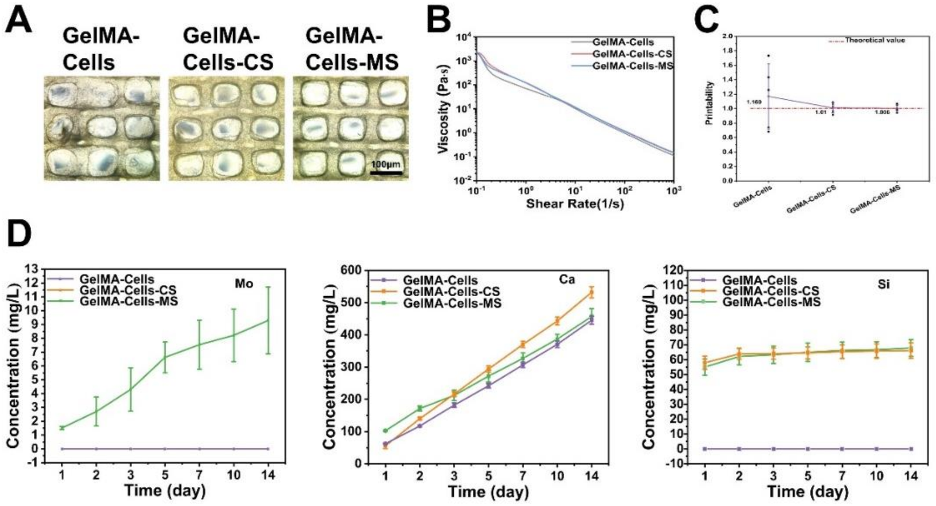

Figure S4. (A) Optical photographs of 3D bioprinted multicellular scaffolds. (B) The shear-thinning properties of bioinks. (C) The printability values of bioinks (n = 6). (D) The ionic release curves of Ca, Mo and Si of 3D bioprinted multicellular scaffolds (n = 3). **The ionic release curves of Ca and Si, rheology and printability of GelMA-Cells-MS were almost identical with those of GelMA-Cells-CS.**

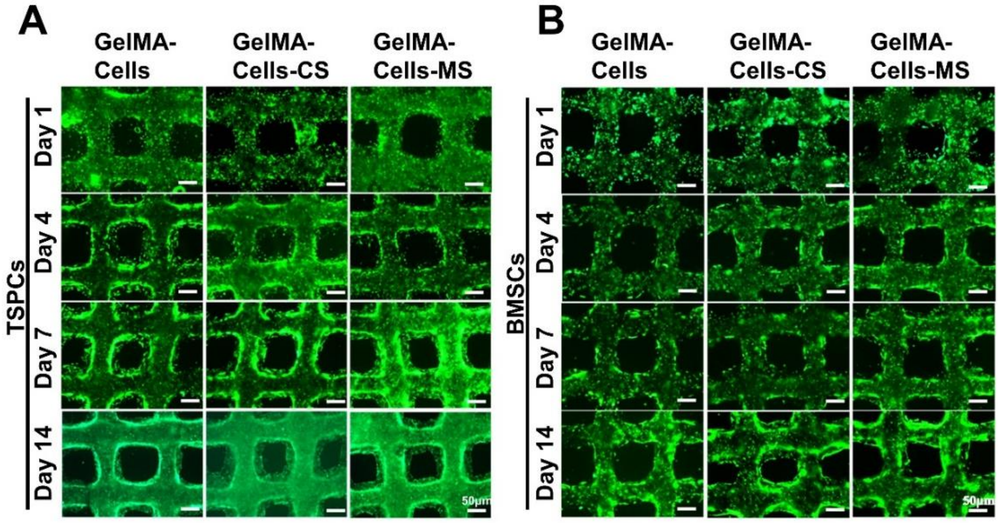

Figure S5. Live/dead assay of (A) TSPCs and (B) BMSCs within the 3D bioprinted multicellular scaffolds on day 1, 4, 7 and 14. **Both of BMSCs and TSPCs were uniformly distributed within the 3D bioprinted multicellular scaffolds and maintained high cell viabilities during the whole culture periods.**

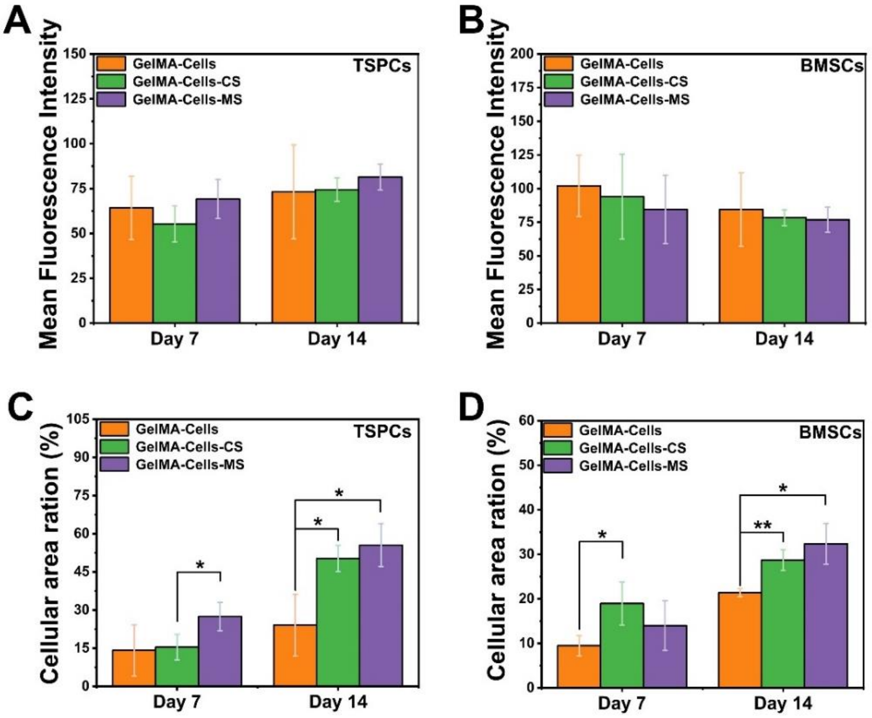

Figure S6. Semi-quantitative analysis of the mean fluorescence intensity of actin expressed by TSPCs (A) and BMSCs (B) on the surface of multicellular scaffolds (n = 3). Semi-quantitative analysis of the area fraction of actin expressed by TSPCs (C) and BMSCs (D) on the surface of multicellular scaffolds (n = 3). *$p < 0.05$, **$p < 0.01$, ***$p < 0.001$. MS and CS bioceramics were beneficial to the migration and diffusion of encapsulated cells.

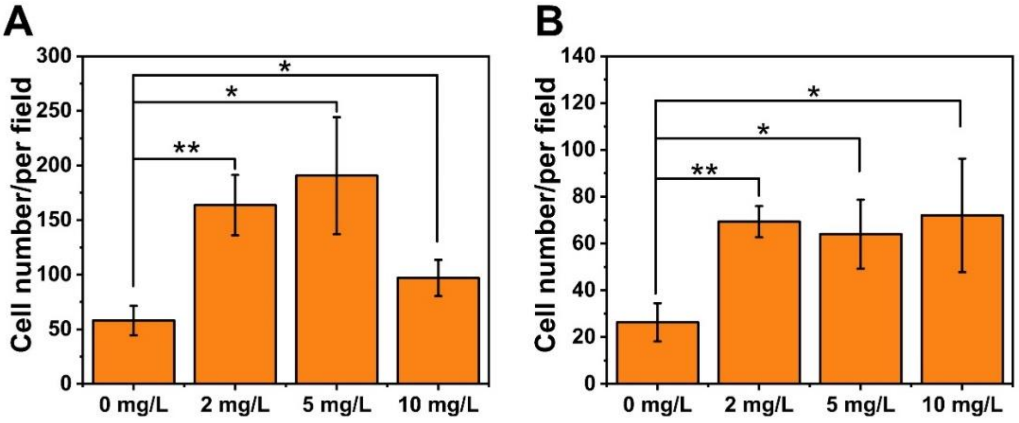

Figure S7. Quantitative statistical analysis of the migrated (A) TSPCs and (B) BMSCs after induced with different concentrations of Mo ions (n = 3). *$p < 0.05$, **$p < 0.01$, ***$p < 0.001$. **Mo ions could promote the migration of TSPCs and BMSCs.**

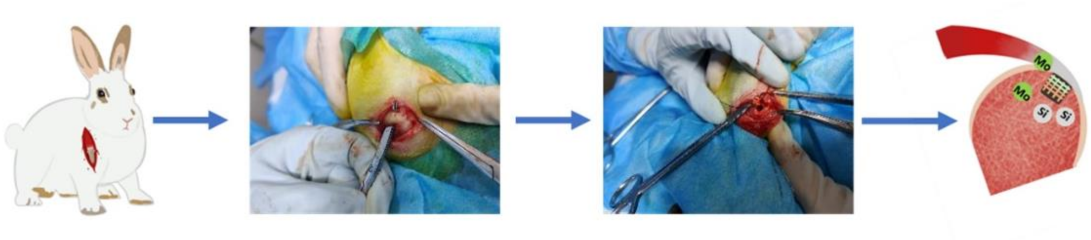

Figure S8. Surgical procedure of rotator cuff tear (RCT) model of rabbits.

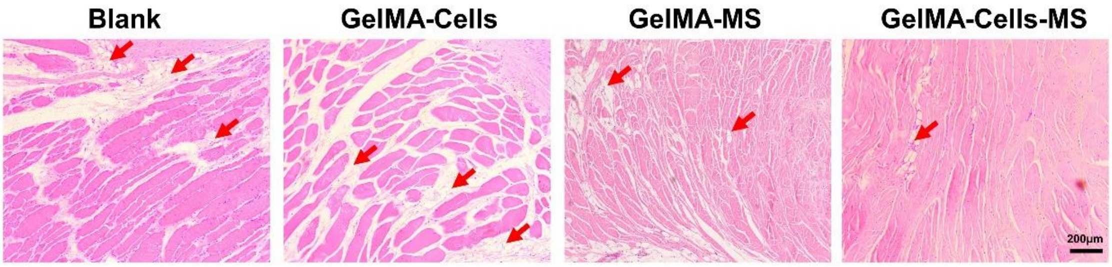

Figure S9. Representative hematoxylin eosin (H&E) staining images of fatty infiltration around the tendon-to-bone interfaces (Red arrows point to fatty infiltration). A large amount of fatty infiltration was observed in Blank and GelMA-Cells groups, while less fatty infiltration was found in the GelMA-MS and GelMA-Cells-MS groups.

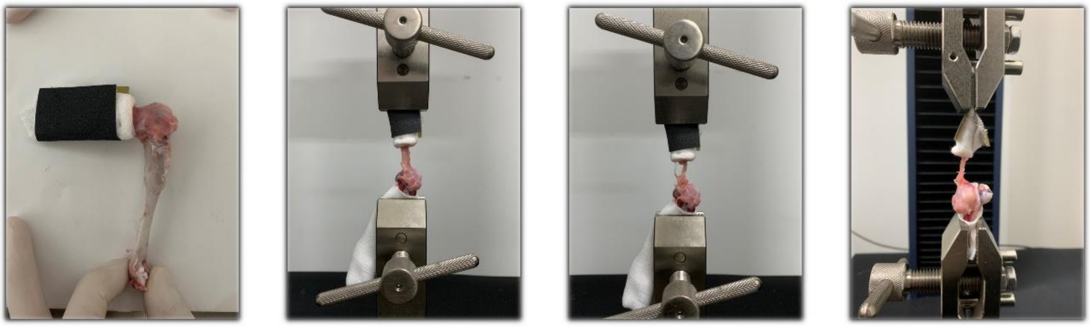

Figure S10. The biomechanical testing procedure of repaired rotator cuffs.

Table S1. The mol ratio of Mo/Ca in Mo-containing silicate bioceramics (*n* = 4).

Mo/Ca(mol%)	Theoretical value	Experiment value
CS	0	0
2Mo-CS	2	1.71
5Mo-CS	5	4.98
10Mo-CS	10	7.55

Table S2. Release behavior of Mo and Si ions from 3D bioprinted single-cell scaffolds during culture (*n* = 4).

Ionic Conc (mg/L)	Time (day)	GelMA	GelMA-1.5MS	GelMA-3MS	GelMA-4.5MS
Mo	1	0.00 ± 0.00	0.29 ± 0.04	1.55 ± 0.12	2.32 ± 0.16
	4	0.00 ± 0.00	0.03 ± 0.05	1.28 ± 0.11	2.32 ± 0.13
	7	0.00 ± 0.00	0.12 ± 0.08	0.88 ± 0.07	1.40 ± 0.19
	10	0.00 ± 0.00	0.07 ± 0.07	0.55 ± 0.07	1.13 ± 0.12
Si	1	0.00 ± 0.00	17.04 ± 3.72	27.19 ± 1.61	44.98 ± 4.00
	4	0.00 ± 0.00	2.09 ± 0.80	4.12 ± 0.34	9.72 ± 1.23
	7	0.00 ± 0.00	0.05± 0.08	0.43 ± 0.05	1.21 ± 0.29
	10	0.00 ± 0.00	0.00 ± 0.00	0.00 ± 0.00	0.09 ± 0.11

Table S3. The primer sequences used for RT-qPCR assays.

Gene		Primer sequence
GAPDH	Forward	TCACCATCTTCCAGGAGCGA
GAPDH	Reverse	CACAATGCCGAAGTGGTCGT
TNC	Forward	CGTGAAAAACAATACCCGAGGC
TNC	Reverse	GCCGTAGGAGAGTTCAATGCC
DCN	Forward	ACTGGGCACCAACCCTCTGA
DCN	Reverse	ATCTGAAGGTGGATGGCTGGA
BGN	Forward	GATGGCCTGAAGCTCAA
BGN	Reverse	GGGTTGTTGAAGAGGCTG
MKX	Forward	AGCGATGACTCGTGTTCCGA
MKX	Reverse	GAGCTCTCGCTTTTGGTCGC
Runx2	Forward	CCTTCCACTCTCAGTAAGAAGA
Runx2	Reverse	TAAGTAAAGGTGGCTGGATAGT
OCN	Forward	CCTCACTCTTGTCGCCCTG
OCN	Reverse	CTCTTGGACACGAAGGCTGA
OPN	Forward	CACCATGAGAATCGCCGT
OPN	Reverse	CGTGACTTTGGGTTTCTACGC
Col1	Forward	GATGGCCTGAAGCTCAA
Col1	Reverse	GGTTTGTTGAAGAGGCTG
BMP2	Forward	CGCCTCAAATCCAGCTGTAAG
BMP2	Reverse	GGGCCACAATCCAGTCGTT

Table S4. The tendon-maturing score criterion.

	Score			
	**1**	**2**	**3**	**4**
Cellularity	Marked	Moderate	Mild	Minimal
Proportion of cells resembling tenocytes	<25%	26-50%	51-75%	>75%
Proportion of cells oriented in parallel	<25%	26-50%	51-75%	>75%
Proportion of fibers of large diameter characteristic of mature tendon fibers	<25%	26-50%	51-75%	>75%
Proportion of fibers orients in parallel	<25%	26-50%	51-75%	>75%
